# An N-terminal antibody promotes the transformation of amyloid fibrils into oligomers and enhances the neurotoxicity of amyloid-beta: the dust-raising effect

**DOI:** 10.1186/s12974-015-0379-4

**Published:** 2015-08-28

**Authors:** Yu-Hui Liu, Xian-Le Bu, Chun-Rong Liang, Ye-Ran Wang, Tao Zhang, Shu-Sheng Jiao, Fan Zeng, Xiu-Qing Yao, Hua-Dong Zhou, Juan Deng, Yan-Jiang Wang

**Affiliations:** Department of Neurology and Centre for Clinical Neuroscience, Daping Hospital and Research Institute of Surgery, Third Military Medical University, 10 Changjiang Branch Road, Yuzhong District, Chongqing, 400042 China

**Keywords:** Alzheimer, Amyloid-beta, N-terminal antibody, Oligomer, Fibril, Dust-raising effect

## Abstract

**Background:**

Senile plaques consisting of amyloid-beta (Aβ) are the major pathological hallmark of Alzheimer’s disease (AD) and have been the primary therapeutic target. Immunotherapies, which are designed to remove brain Aβ deposits, increased levels of soluble Aβ and accelerated brain atrophy in some clinical trials, suggesting that the solubilization of Aβ deposition might facilitate the formation of more toxic Aβ oligomers and enhance neurotoxicity.

**Methods:**

The capacity of antibodies against different epitopes of Aβ to disaggregate preformed Aβ fibrils was investigated. The co-incubation of antibodies and Aβ fibrils was then tested for neurotoxicity both *in vitro* and *in vivo*.

**Results:**

After the incubation of preformed Aβ fibrils with the N-terminal antibody 6E10, the fibrils were decreased, while the oligomers, mostly dimers and trimers, were significantly increased. However, no such effects were observed for antibodies targeting the middle domain (4G8) and C-terminus of Aβ (8G7). The co-incubates of preformed Aβ fibrils with 6E10 were more neurotoxic, both in vitro and in vivo, than the co-incubates with 4G8 and 8G7.

**Conclusions:**

Our results indicate that the antibody targeting the N-terminus of Aβ promoted the transformation of Aβ from fibrils into oligomers and increased neurotoxicity. Immunotherapies should take into consideration the enhanced neurotoxicity associated with the solubilization of Aβ deposits by antibodies against the Nterminus of Aβ.

## Introduction

Senile plaque containing amyloid-beta (Aβ) protein are a major hallmark of Alzheimer’s disease (AD) and have been considered as an important therapeutic target of AD [1, 2]. Immunotherapies are promising for the treatment of AD by removing senile plaques and attenuating the pathologies secondary to Aβ, such as tau pathologies, neuroinflammation, dendritic dysfunction and neuronal loss [3]. However, current clinical trials of immunotherapies for AD failed to improve cognition and reverse disease progression. Several significant adverse effects were observed in AD clinical trials, including meningoencephalitis, vasogenic oedema and microhaemorrhage [4]. Some immunotherapies could not reduce or even increased the levels of soluble Aβ in both animal and clinical trials [5, 6]. It was suggested that oligomers are the most toxic form of Aβ aggregates [7]. In this regard, the transformation of Aβ oligomers into fibrils might be an adaptive change in AD; disaggregation Aβ fibrils into oligomers in immunotherapies may enhance the neurotoxicity of this peptide. This might explain the failure of AN1792 clinical trials in which alleviation of the brain amyloid burden was accompanied by accelerated brain atrophy and the deterioration of cognitive function in patients receiving the vaccine [8]. In the present study, we aimed to investigate the capacity of antibodies targeting different epitopes of Aβ to disaggregate preformed Aβ fibrils and to determine whether antibody-induced disaggregation of Aβ fibrils can facilitate the formation of oligomers and enhance the neurotoxicity of Aβ.

## Materials and methods

### Preparation of Aβ fibrils

Synthetic Aβ42 was purchased from American Peptide (CA, USA). Aβ fibrils were prepared according to previous protocols [9]. Briefly, Aβ1–42 was dissolved in 100 μL of ice-cold Dulbecco’s modified Eagle’s medium (DMEM, Gibco) (pH = 7.5) containing 0.05 % NaN_3_. Solutions containing 10 μg of Aβ42 were incubated at 37 °C for 72 h for polymerization. The reaction tubes were not agitated during the reaction. After incubation, the mixture was centrifuged at 4 °C for 20 min at 8000×*g*. The supernatant was discarded, and the precipitation was resuspended in 50 μL of phosphate-buffered saline (PBS) containing 0.05 % NaN_3_ in an Eppendorf tube and stored at 4 °C for further use.

### Preparation of antibody-Aβ co-incubates

The monoclonal antibodies used in the present study, including 6E10 (antigen epitope, Aβ residues 1–17; affinity to Aβ monomer, 22.3 nM [10]; Covance), 4G8 (antigen epitope, Aβ residues 17–24; affinity to Aβ monomer, 30.1 nM [10]; Covance) and 8G7 (antigen epitope, Aβ residues 41–42; Acris), were dissolved in distilled water at a concentration of 1 μg/μL. Preformed Aβ fibrils generated from 10 μg of Aβ42 were resuspended in 9 μL of distilled water, followed by the addition of 1 μL of antibody solution. Aβ fibril suspensions with 1 μL PBS were incubated under the same conditions as a control. The mixture was incubated at 37 °C for 72 h before the assay.

### Thioflavin T assay

To test whether antibodies, including 6E10, 4G8 and 8G7, can disaggregate Aβ fibrils, Aβ42 (10 μM) was dissolved in distilled water and incubated in 96-well plates at 37 °C for 48 h for fibrillization. Antibodies (2 μg in 1 μL) were then added to the wells, and the samples were incubated at 37 °C for another 72 h. Aβ42 monomers were incubated with PBS under the same conditions as the negative control. The samples were then measured by adding 5 μM thioflavin T (ThT) solution (50 mM phosphate buffer, pH 6.0). Fluorescence intensity was monitored at an excitation wavelength of 450 nm and an emission wavelength of 482 nm by a spectrometer (Synergy H4, Bio Tek). Each experiment was performed in triplicate, and the means of the triplicates were used for the statistical analysis.

### Western blot

A 10-μL aliquot of the co-incubation samples as described above was loaded onto SDS-PAGE gradient (4 %-10 %-16 % acrylamide) gels. Separated Aβs were transferred to nitrocellulose membranes. The blots were probed with biotin-conjugated 6E10. Infrared dye-conjugated streptavidin (Li-COR Biosci, NE) was used to detect positive bands. The membranes were visualized with an Odyssey Imaging System (Odyssey V3.0). The density was calculated with western blot analysis software (Quantity One V4.62).

### Transmission electron microscopy negative staining

To validate the effect of antibodies on the disaggregation of Aβ fibrils, transmission electron microscopy (TEM) negative staining was performed. A 10-μL aliquot of preformed Aβ42 fibrils that were co-incubated with antibodies (as prepared above) was spotted onto a glow-discharged, carbon-coated Formvar grid and incubated for 20 min at room temperature. The droplet then was displaced with an equal volume of 2.5 % (*v*/*v*) glutaraldehyde and incubated for an additional 6 min. Finally, the peptide was stained with 2 % aqueous phosphotungstic acid for 30 s. Samples were examined using a Joel 1200 EX transmission electron microscope equipped with a Megaview 3 digital camera. The area of fibrils was selected for automatic quantification using Image J software, and the analysis yielded the fractional area of the total positive staining against the area of the analysed field.

### Annexin V labelling

An annexin V staining kit (KeyGEN BioTECH) was used according to the manufacturer’s instructions to evaluate the proportion of apoptotic cells. Briefly, SH-SY5Y cells were seeded at a density of 10^5^ cells/mL in 12-well plates (Sarstedt) in DMEM (Gibco) containing 10 % foetal bovine serum (FBS, Gibco) at 200 μL per well. The co-incubates (20 μL) of different antibodies or PBS were added to the wells. After 3 h of incubation, live cells were washed three times with warm PBS for 5 min. Then, they were treated with FITC-labelled annexin V for 5 min at room temperature and subsequently washed with PBS. The cells were then incubated with DAPI (1:1000) for 5 min at room temperature and washed with PBS. Cell images were collected with a B50 fluorescence microscope.

### MTT assays

The neurotoxicity of the co-incubation samples was measured using an MTT (3-(4,5-dimethylthiazol-2-yl)-2,5-diphenyl tetrazolium bromide) viability assay as previously described [11]. Briefly, SH-SY5Y cells were seeded at a density of 10^5^ cells/mL in a 96-well plate in DMEM containing 10 % FBS at 50 μL per well. The co-incubates (10 μL) were added to the wells. After 20 h of incubation, 10 μL of MTT (Sigma-Aldrich, USA, 5 mg/mL in PBS) was added to each well, and the samples were incubated for 4 h. A solubilization solution (10 % SDS in 0.01 M hydrochloric acid) was added to dissolve the insoluble purple formazan product to produce a coloured solution. Each assay was performed in triplicate. The optical density (OD) was read at 600 nm on a multi-well scanning spectrophotometer (BIO-RAD Model 2550 EIA Reader).

### Neurite outgrowth assay

For the neurite outgrowth assays, SH-SY5Y cells were cultured for 7 days in a medium with 1 % FBS and 10 μM all-trans-retinoic acid (RA) (Sigma, USA) and then incubated with 2 μL of the co-incubates for 24 h. Each assay was performed in triplicate. The cell images were taken by microscopy, and the length of 10 neurites per view field were measured. Data from 20 view fields per group were analysed.

### Mouse and brain injections of antibody-Aβ co-incubates

Six-month-old C57BL/6J mice were housed and maintained in the animal facility at Daping Hospital. We used only females in our analyses (*n* = 5 for each antibody group). A midsagittal incision was made to expose the cranium, and a burr hole was drilled with a dental drill over the left hemisphere to the following coordinates: anteroposterior, −0.2 mm; lateral, 1 mm; and ventral, 2.2 mm, which were all taken from the bregma. Co-incubates (5 μL) were injected into the lateral ventricle of the mice. PBS (5 μL) was injected in the same manner as a control. Forty-eight hours after injection, the brains were fixed and sectioned. Five sections around the injection site were selected per animal. This study and all experimental protocols were approved, and the methods were carried out in accordance with the guidelines of the Animal Care Committee of the Third Military Medical University (TMMU).

### Analysis of neuronal apoptosis

To detect apoptotic cells, terminal deoxynucleotidyl transferase dUTP nick end labelling (TUNEL) staining and immunofluorescence with anti-activated caspase-3 were used. For TUNEL staining, apoptotic cells were labelled using an in situ Death Detection Kit, POD (Roche), according to the manufacturer’s instructions. The sections were also detected with NeuN and caspase-3 double-labelling immunofluorescence. The area of the hippocampus was selected for automatic quantification using Image J software to detect NeuN and caspase-3 positive neurons, yielding the area fraction of the total positive staining against the area of analysed tissue. The average of the individual measurements was used to calculate group means and standard error of mean (SEM). The quantification of the area fraction and positive cells was performed using ImageJ.

### Statistical analysis

The results were presented as the mean ± SEM. The data were first assessed for normal distribution by the one-sample Kolmogorov-Smirnov test. Statistical comparisons among groups were tested using one-way ANOVA. Two-way ANOVA was used to compare the antibody-induced time-dependent disaggregation of Aβ fibrils in the ThT assay. *P* values <0.05 were considered statistically significant.

## Results

### Antibody targeting the N-terminus of Aβ promoted the disaggregation of preformed Aβ fibrils

**Fig. 1:**
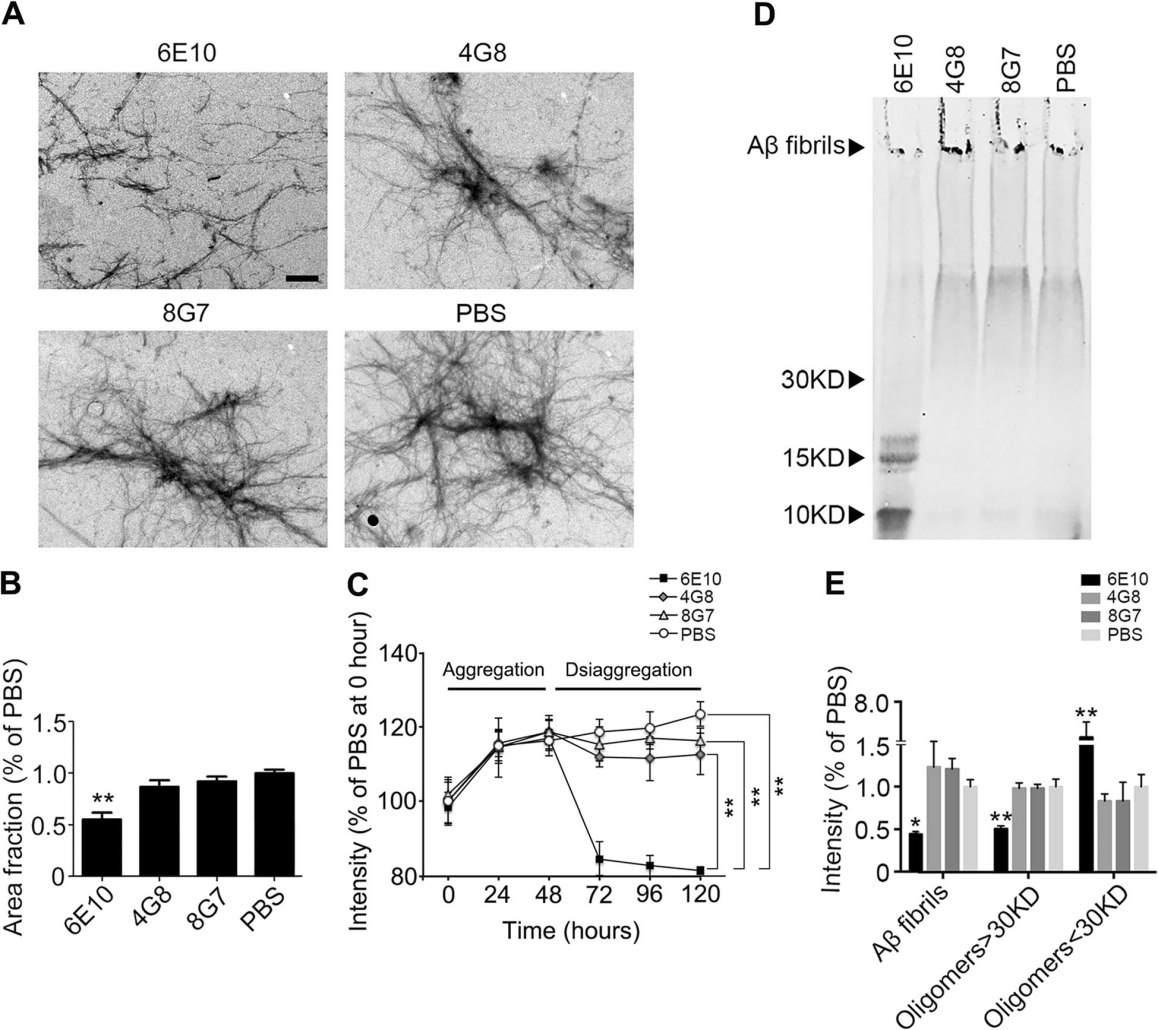
An N-terminal antibody promotes the disaggregation of preformed Aβ fibrils. **a** Representative TEM images of Aβ fibrils after treatment with antibodies targeting different sequences of Aβ. **b** Quantification of the fibrillar area fractions of TEM images. **P < 0.01 vs PBS. **c** Fluorescence intensity of the ThT assays showing the capacity of antibodies to disaggregate preformed Aβ fibrils. Aβ42 monomers were aggregated for 48 h and were then incubated with antibodies or PBS for an additional 72 h for disaggregation. **P < 0.01. **d** Western blot image showing the capacity of antibodies to disaggregate preformed Aβ fibrils. **e** Quantification of the relative intensity of western blot bands. **P < 0.01 vs control, #*P* < 0.05 and ##*P* < 0.01 vs PBS. *TEM* transmission electron microscopy, *ThT* thioflavin T

TEM and ThT assays were conducted to investigate the capacity of antibodies to disaggregate preformed Aβ fibrils. After the incubation of preformed Aβ fibrils with N-terminal antibody 6E10, which targets amino acids 1–16 of Aβ, truncated fibrils and small aggregates of a variable size were observed in the TEM assay (Fig. 1a). However, after the incubation with 4G8, which targets amino acids 17–24 of Aβ, and 8G7, which targets the C-terminus of Aβ and PBS-treated samples, highly aggregated fibrils were observed under TEM. This variance in the disaggregation ability of the antibodies was indicated by significantly lower area fractions of Aβ fibrils in the 6E10 group (*P* = 0.001 vs PBS) (Fig. 1b).

The ThT assay was conducted to further investigate the time-dependent disaggregation of Aβ fibrils induced by antibodies. Aβ42 monomers were aggregated for 48 h and then co-incubated with antibodies for an additional 72 h for disaggregation. We found that the fluorescence intensity induced by Aβ fibrils was significantly lower in 6E10-treated samples compared to 4G8- and 8G7-treated samples and the PBS controls (Fig. 1c).

These data suggest that an antibody against the N-terminus of Aβ was able to disaggregate Aβ fibrils.

### Antibody targeting the N-terminus of Aβ promoted the transformation of Aβ fibrils to oligomers

We next used western blot to detect whether disaggregation of Aβ fibrils could facilitate the formation of Aβ oligomers. After the incubation of preformed Aβ fibrils with antibodies, the co-incubates were subjected to western blotting. We found that more dimers and trimers (*P* < 0.001 vs PBS) and fewer fibrils (*P* = 0.001 vs PBS) and aggregates with a molecular weight more than 30 kDa (*P* = 0.016 vs PBS) were observed in the 6E10-treated samples compared with the 4G8- and 8G7-treated samples (Fig. 1d, e). There was no difference in the amount of Aβ oligomers among the 4G8/8G7-treated samples and PBS controls. These findings suggest that an antibody against the N-terminus of Aβ facilitated the transformation of Aβ fibrils into Aβ oligomers.

### Antibody targeting the N-terminus of Aβ increased the neurotoxicity of Aβ *in vitro*

**Fig. 2:**
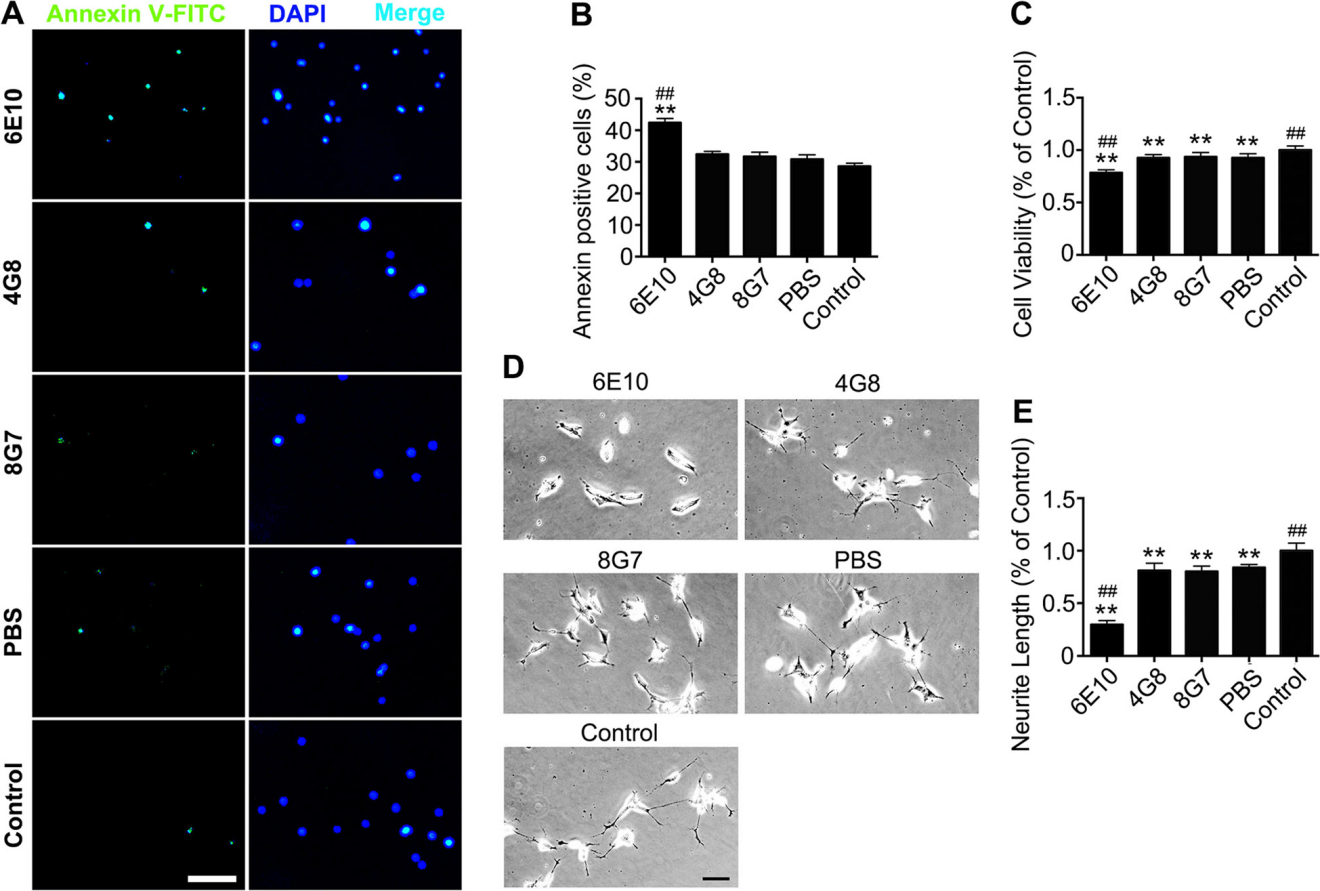
Disaggregation of preformed Aβ fibrils by an N-terminal antibody increases the neurotoxicity of Aβ in vitro. **a** Representative images of annexin V staining in SH-SY5Y cells treated with the co-incubates of antibodies and Aβ. The neurons were counterstained with DAPI. Scale bar = 100 μm. **b** Quantification of the percentages of annexin V-labelled neurons. **c** MTT assays showing the cell viability of SH-SY5Y cells. **d** Representative images of SH-SY5Y cells treated with co-incubates of antibodies and Aβ. **e** Quantification of the neurite length of SH-SY5Y cells. **P < 0.01 vs control, ##*P* < 0.01 vs PBS

Based on the above findings, we proposed that the disaggregation of preformed fibrils might increase the toxicity of Aβ by facilitating the formation of Aβ oligomers. Annexin V staining, neurite outgrowth and MTT assays were conducted to investigate the neurotoxicities of antibody-fibril co-incubates in vitro. Interestingly, the N-terminal antibody 6E10 significantly increased the neurotoxicity of Aβ fibrils as reflected by the significant increase in the percentage of annexin V-labelled cells (Fig. 2a, b), the decrease in cell viability (Fig. 2c) and the neurite length (Fig. 2d, e) of SH-SY5Y cells. However, the percentage of annexin V-labelled cells, cell viability and neurite length were comparable among the 4G8, 8G7 and PBS groups (Fig. 2a–c).

### Antibody targeting the N-terminus of Aβ increased the neurotoxicity of Aβ *in vivo*

**Fig. 3:**
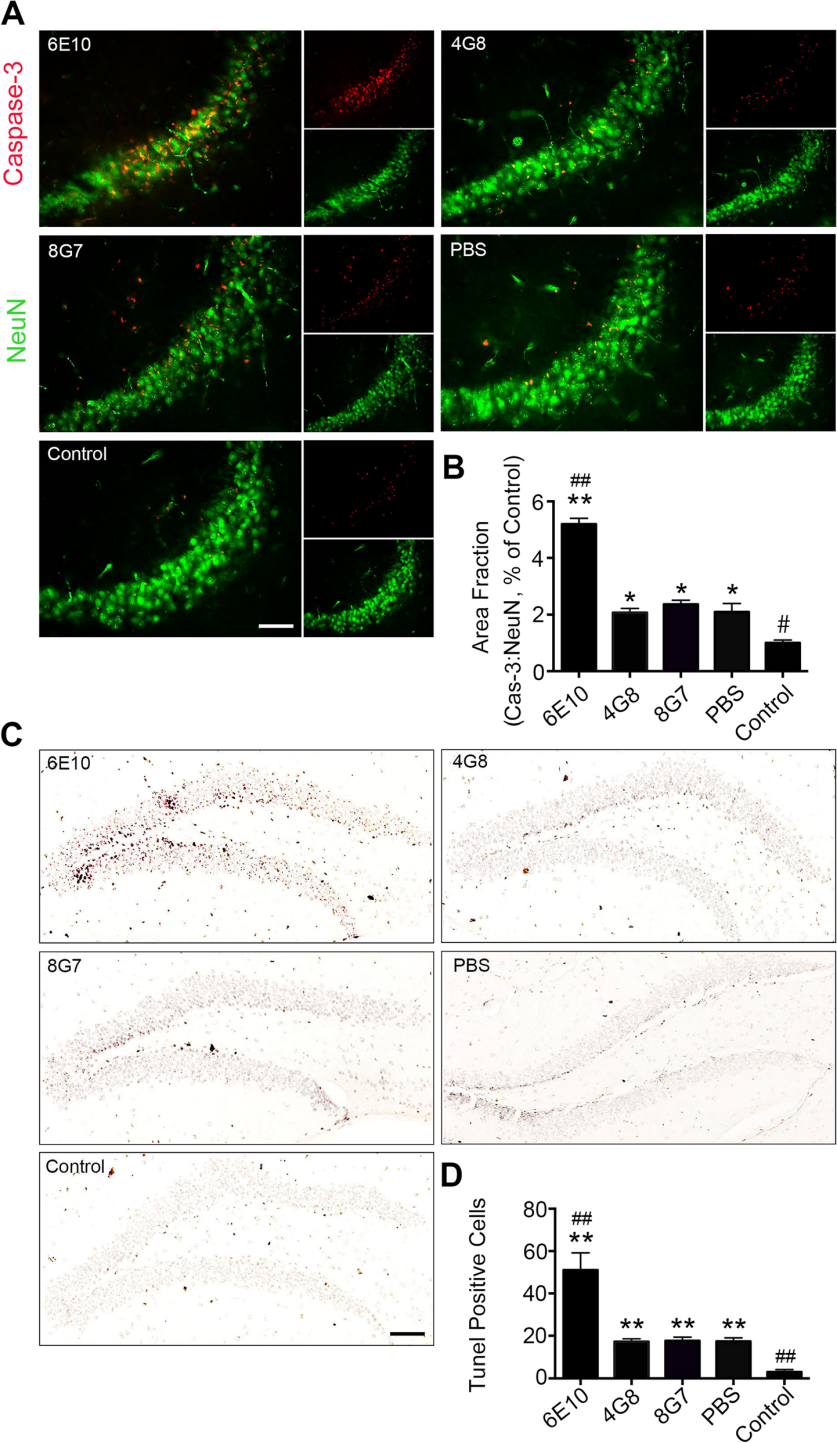
Disaggregation of preformed Aβ fibrils by an N-terminal antibody increases the neurotoxicity of Aβ *in vivo*. **a** Neuronal apoptosis in the CA3 region was visualized using NeuN and activated caspase-3 co-staining (scale bar = 50 μm). **b** Quantification of apoptosis by activated caspase-3 in the CA3 region. **c** Neuronal apoptosis in the dendrite gyrus was visualized using TUNEL staining (scale bar = 200 μm). **d** Quantification of apoptotic cells in the dendrite gyrus that were stained by TUNEL (*n* = 5 per group, mean ± SEM, One-way Anova,*P < 0.05 and **P < 0.01 vs control, #*P* < 0.05 and ##*P* < 0.01 vs PBS)

We further investigated the neurotoxicity of Aβ fibril-antibody co-incubates *in vivo*. After the injection of co-incubates into the lateral ventricle of 6-month-old C57 mice in each group, we conducted NeuN and caspase-3 double staining, as well as TUNEL staining, to investigate the neurotoxicity in vivo. Consistent with the *in vitro* results, co-incubates in the 6E10 group significantly induced neuronal apoptosis in both the CA3 region (*P* < 0.001 vs PBS; *P* < 0.001 vs control) (Fig. 3a, b) and the dendrite gyrus (*P* < 0.001 vs PBS; *P* < 0.001 vs control) (Fig. 3c, d). However, no significant difference was found in neuronal apoptosis among the 4G8, 8G7 and PBS groups (Fig. 3a–d).

## Discussion

In the present study, for the first time, we found that an antibody against the N-terminus of Aβ, but not antibodies against the middle domain or the C-terminus of Aβ, disaggregated preformed Aβ fibrils, leading to the formation of oligomers and enhancing the neurotoxicity of Aβ both *in vitro* and *in vivo*.

Immunotherapies are promising for the treatment of AD by reducing amyloid deposition and improving cognition in animal models of AD. The mechanisms of the antibody-mediated Aβ clearance included solubilization of Aβ fibrils [12, 13], antibody-mediated phagocytosis of Aβ by microglia [14, 15] and sequestration of Aβ in the blood as a peripheral “sink” [16]. However, despite their effects in reducing brain Aβ deposition, immunotherapies have not been successful in improving cognition in clinical trials [17–20]. Although these failures are mainly attributed to the fact that interventions occurred too late to reverse the disease, the adverse effects associated with immunotherapies including autoimmune meningoencephalitis, vasogenic oedema and microhaemorrhage are also important factors that compromise the therapeutic efficacy of immunotherapy [4]. An important phenomenon derived from previous clinical and experimental studies is that immunotherapies are effective in reducing brain amyloid deposition but cannot reduce and sometimes even increase the levels of soluble Aβ [5, 6, 21]. The increase in soluble Aβ is a result of the solubilization of Aβ fibrils by antibodies. Convincing data has arisen suggesting that the soluble oligomeric Aβ species are the primary toxic agents in AD [7]. Whether transformation of deposited Aβ plaques into soluble Aβ can favour the formation of Aβ oligomers remains unknown. We have previously proposed that solubilization of Aβ deposits might favour the formation of more toxic Aβ oligomers, thus enhancing the neurotoxicity of Aβ in immunotherapies; we have termed this phenomenon as the “dust-raising effect” [4]. In the present study, we found that an antibody against the N-terminus of Aβ (6E10) promoted the transformation of Aβ fibrils into toxic oligomers, primarily Aβ dimers and trimers, which are the major toxic forms of Aβ [22, 23] that cause significant neuronal death in the brain of mice. This finding is of significant clinical relevance. In the AN1792 trial, brain Aβ deposition was removed; however, the soluble Aβ species were elevated, and brain volume loss was accelerated [24]. The reason for this dissociation between Aβ clearance and brain atrophy remains unclear. It was proposed that the volume changes were due to amyloid removal and associated cerebral fluid shifts [24]. According to our present findings, it is likely that the oligomeric Aβ species derived from the solubilization of Aβ deposits caused further damage to neurons in the brain, leading to the subsequent acceleration of brain volume loss.

In addition, the adverse effects of immunotherapies were also associated with the epitopes of the Aβ N-terminus. Vasogenic oedema and microhaemorrhage were observed in passive immunotherapies utilizing antibodies against the N-terminus of Aβ [17] but not antibodies against the middle domain of Aβ [18]. The possible reason for this is that antibodies bind to the N-terminus of Aβ, which is exposed on the surface of Aβ fibrils, and form immune complexes that induce subsequent inflammatory reactions [4, 25]. We also found that antibodies to the N-terminus of Aβ can promote the generation of Aβ by cross-reacting with neurons via the N-terminal epitope of Aβ, which is located in the extracellular domain of APP and is exposed on the surface of neurons [26, 27].

In our present study, we found that an antibody targeting the N-terminus of Aβ, but not antibodies to the middle domain and C-terminus of Aβ, was able to disaggregate Aβ fibrils, suggesting that the therapeutic function of anti-Aβ antibodies are closely related to their antigen epitopes. Conformational analysis showed that the N-terminus of Aβ was exposed on the surface, but the middle domain and C-terminus were embedded in the core of Aβ fibrils [28]. Thus, N-terminal antibodies can potently clear Aβ plaque because they are accessible to Aβ fibrils, but antibodies to the middle domain and C-terminus of Aβ may have limited potential to recognize preformed Aβ fibrils and are therefore less effective as a treatment to remove Aβ deposits. This notion was supported by the fact that solanezumab, an antibody against the middle domain of Aβ, is not as effective in reducing the brain Aβ burden [18] compared to bapineuzumab, an antibody against the N-terminus of Aβ that is able to remove brain Aβ deposits, as evidenced by recent immunotherapeutic clinical trial [17, 29]. It was revealed that antibodies targeting the middle domain of Aβ have a better anti-aggregation capacity than those targeting the N-terminus [30]. The competency of these antibodies in antagonizing Aβ aggregation can be explained by the findings that the middle domain of Aβ is key for Aβ aggregation [31]. In this regard, antibodies against the middle domain of Aβ might be more effective as a preventative treatment before the deposition of Aβ.

In conclusion, the present study indicated that solubilization of Aβ fibrils by an antibody against the N-terminus of Aβ leads to the formation of more toxic Aβ oligomeric species. The therapeutic and adverse effects of anti-Aβ antibodies are associated with their antigen epitopes. This should be taken into consideration for the efficacy and safety of immunotherapies in the future.
